# The envelope protein of tick-borne encephalitis virus influences neuron entry, pathogenicity, and vaccine protection

**DOI:** 10.1186/s12974-020-01943-w

**Published:** 2020-09-28

**Authors:** Richard Lindqvist, Ebba Rosendal, Elvira Weber, Naveed Asghar, Sarah Schreier, Annasara Lenman, Magnus Johansson, Gerhard Dobler, Malena Bestehorn, Andrea Kröger, Anna K. Överby

**Affiliations:** 1 Department of Clinical Microbiology, Section of Virology, Umeå University, Umeå, Sweden; 2 The Laboratory for Molecular Infection Medicine Sweden (MIMS), Umeå, Sweden; 3 Current affiliation: Life & Medical Sciences Institute (LIMES), University of Bonn, Bonn, Germany; 4 School of Medical Sciences, Inflammatory Response and Infection Susceptibility Centre (iRiSC), Faculty of Medicine and Health, Örebro University, Örebro, Sweden; 5 Institute of Medical Microbiology, Otto-von-Guericke-University Magdeburg, Magdeburg, Germany; 6 Innate Immunity and Infection, Helmholtz Centre for Infection Research, Braunschweig, Germany; 7 Institute for Experimental Virology, TWINCORE, Centre for Experimental and Clinical Infection Research, a joint venture between the Medical School Hannover and the Helmholtz Centre for Infection Research, Hannover, Germany; 8 Bundeswehr Institute of Microbiology, Munich, Germany; 9 Parasitology Unit, University of Hohenheim, D-, Stuttgart, Germany

**Keywords:** Tick-borne encephalitis virus, European subtype, Envelope protein, Pathogenesis, Neurovirulence

## Abstract

**Background:**

Tick-borne encephalitis virus (TBEV) is considered to be the medically most important arthropod-borne virus in Europe. The symptoms of an infection range from subclinical to mild flu-like disease to lethal encephalitis. The exact determinants of disease severity are not known; however, the virulence of the strain as well as the immune status of the host are thought to be important factors for the outcome of the infection. Here we investigated virulence determinants in TBEV infection.

**Method:**

Mice were infected with different TBEV strains, and high virulent and low virulent TBEV strains were chosen. Sequence alignment identified differences that were cloned to generate chimera virus. The infection rate of the parental and chimeric virus were evaluated in primary mouse neurons, astrocytes, mouse embryonic fibroblasts, and in vivo. Neutralizing capacity of serum from individuals vaccinated with the FSME-IMMUN® and Encepur® or combined were evaluated.

**Results:**

We identified a highly pathogenic and neurovirulent TBEV strain, 93/783. Using sequence analysis, we identified the envelope (E) protein of 93/783 as a potential virulence determinant and cloned it into the less pathogenic TBEV strain Torö. We found that the chimeric virus specifically infected primary neurons more efficiently compared to wild-type (WT) Torö and this correlated with enhanced pathogenicity and higher levels of viral RNA in vivo. The E protein is also the major target of neutralizing antibodies; thus, genetic variation in the E protein could influence the efficiency of the two available vaccines, FSME-IMMUN® and Encepur®. As TBEV vaccine breakthroughs have occurred in Europe, we chose to compare neutralizing capacity from individuals vaccinated with the two different vaccines or a combination of them. Our data suggest that the different vaccines do not perform equally well against the two Swedish strains.

**Conclusions:**

Our findings show that two amino acid substitutions of the E protein found in 93/783, A83T, and A463S enhanced Torö infection of neurons as well as pathogenesis and viral replication in vivo; furthermore, we found that genetic divergence from the vaccine strain resulted in lower neutralizing antibody titers in vaccinated individuals.

## Background

Tick-borne encephalitis virus (TBEV) is a positive-sense, single-stranded RNA virus belonging to the family of Flaviviridae and genus *Flavivirus*. In Europe, TBEV is considered to be the most important arthropod-borne viral infection and causes about 10,000–13,000 cases of TBE worldwide every year [1, 2]. TBEV is most commonly transmitted by the bite of an infected tick, but transmission by consumption of unpasteurized dairy products [3, 4] as well as via solid organ transplantation may occur [5]. The symptoms of a TBEV infection range from a subclinical form to a mild febrile illness, to meningitis and encephalitis. The exact determinants of the disease severity are not known; however, infectious dose, age, genotype, and immune status of the host as well as the virulence of the virus strain are thought to play a role [6–8].

Based on phylogenetic differences, TBEV is divided into three different subtypes: European, Siberian, and Far Eastern [9]. At least two more subtypes, the Baikalian and the Himalayan subtypes, are proposed [10]. The genetic difference on the amino acid level is up to 2% within a subtype and 5–6% in between subtypes [11]. In general, the European subtype is associated with milder disease and lower case fatality rates (0.5–2%) compared to the Siberian and Far Eastern subtypes (fatality rates between 5 and 20%) [12, 13]. However, both mild and severe cases have been observed with all three subtypes [14].

Once infected, there are no specific antiviral therapies approved in Europe against TBEV so treatment is restricted to supportive care and symptomatic therapy. However, there are two commercially available vaccines in Europe, FSME-IMMUN® and Encepur®, which are considered to be safe and efficacious [15, 16]. Although the vaccine is considered to be very efficient, vaccine breakthroughs have occurred despite complete vaccination [17–20]. Previous studies have shown that serum from vaccine breakthrough patients contained high titers of antibodies able to neutralize the vaccine strain Neudörfl (FSME-IMMUN®) and the closely related strain Hypr [17, 21]. Furthermore, it was shown that neutralizing titers decreased with increased genetic diversity from the Hypr strain [21]; thus, genetic variance in natural isolates might contribute to vaccine breakthroughs.

Not much is known about the viral determinants responsible for disease severity in TBEV infection, since the virus is often undetectable in TBE patients, thus making the correlation between virus strain and disease severity difficult. Previous studies have shown that cell culture adaptations of TBEV have led to amino acid changes in the envelope (E) protein resulting in stronger viral interactions with glycosaminoglycans, which was accompanied with attenuated neuroinvasiveness [22, 23]. But so far, no amino acid substitutions in the E protein have been found to increase TBEV infection of the main target cell in the brain, the neurons.

Here we wanted to identify novel virulence determinants in TBEV infection. We infected mice with nine different TBEV strains. We identified 93/783 (European subtype) as a highly neurovirulent and pathogenic TBEV strain compared to strains from the European, Siberian, and Far Eastern subtypes. After sequence alignment, we found two unique amino acid residues in the E protein of 93/783 that were cloned into the infectious clone of Torö (based on the Torö-2003 strain, European subtype). We found that transfer of the E protein of 93/783 into Torö enhanced the infection rate of neurons and enhanced the pathogenesis in mice. Compared to the vaccine strains of TBEV, these strains differ in regions of the E protein known to be involved in neutralization. Therefore, we investigated the neutralizing capacity of sera from people vaccinated with either FMSE-IMMUN®, Encepur®, or a combination of both vaccines against these viruses. In general, we found lower neutralizing antibody titers against all tested strains in the Encepur® group compared to the other two groups. Furthermore, FSME-IMMUN® and the combined vaccine groups showed higher titers against Neudörfl compared to 93/783 and Torö strains.

## Methods

### Ethic statement

Infection experiments with animal were performed in compliance with the German animal welfare law (TierSchG BGBl. S. 1105; 25.05.1998) at the Helmholtz Center for Infection Research in Braunschweig. The mice were housed and handled in accordance with good animal practice as defined by FELASA. All animal experiments were approved by the Lower Saxony State Office of Consumer Protection and Food Safety under permit number AZ 33.19-42502-04-15/1895. For primary cell isolation, mice were maintained under specific pathogen-free conditions and experiments were approved and carried out according to the guidelines set out by the regional animal ethical committee (Umeå, Sweden) under approval number A77-14. For the serological analysis, the research was carried out in line with “The Code of Ethics of the World Medical Association” (Declaration of Helsinki) and according to good clinical practice guidelines. In accordance with local legislation, no formal approval by a research ethics committee was required, because either anonymous samples or sera for research purposes were used.

### Viruses and cells

VeroB4 cells were cultured in medium 199/EBSS (Hyclone) supplemented with 10% FBS (Hyclone), 100 U/mL of penicillin, and 100 μg/mL streptomycin (Gibco). Astrocytes and neurons were isolated and cultured as previously described [24, 25]. In brief, astrocytes were isolated from cerebral cortices between postnatal days 1 and 4, seeded on poly-d-lysine (Sigma) coated T75 flasks, and cultured in DMEM (Sigma) supplemented with 10% heat-inactivated FBS (Hyclone), 100 U/mL of penicillin and 100 μg/mL streptomycin (Gibco), and 2 mM l-glutamine (Gibco). Neurons were isolated at embryonic day 17 and seeded on poly-d-lysine coated plates and cultured in neurobasal medium supplemented with B27 (Gibco), 100 U/mL of penicillin and 100 μg/mL streptomycin (Gibco), and 2 mM l-glutamine (Gibco). 93/783 virus strain was isolated from the serum of a 56-year-old male in Sweden. Serum samples were taken 5 days post onset of symptoms and the patient later developed a biphasic course of the disease [26]; the virus was a kind gift of S. Vene (Folkhälsoinstitutet, Stockholm, Sweden). All virus stocks were grown and titrated on VeroB4 cells.

### Viral infection of mice

C57BL/6 mice were bought from Harlan laboratories/ENVIGO. Six- to 10-week-old mice were intraperitoneally infected with the 10^4^ focus-forming units (FFU) of the indicated strain of TBEV in 100 μL phosphate-buffered saline (PBS). For intracranial infections, mice were anesthetized by intraperitoneal injection with a mixture of ketamine (100 μg/g body weight) and xylazine (5 μg/g body weight). Mice were then injected with 10^2^ FFU of the indicated strain of TBEV in 20 μl PBS. Each mouse was scored daily for clinical signs of disease and humanely killed when reaching an accumulative score of 2 (0, no clinical signs; 1, weight decrease ≥ 10%, decrease grooming, slight uncoordinated movements, slight automutilation, slight exhausting breathing; 2, weight decrease ≤ 20%, unregular fur, uncoordinated movements, automutilation, exhausting breathing). For organ harvesting, mice were perfused with 20 mL of PBS to remove blood from the tissue and organs were harvested in Trizol reagent for RNA extraction. Experiments were performed in the biosafety level 3 (BSL3) facility at the Helmholtz Center for Infection Research.

### Rescue and cloning of chimeric virus

Generation of a chimeric clone containing E gene of 93/783 in the Torö background. The infectious clone was constructed using two plasmids: pTBEV-CME and pTBEV-*luc-*rep for Torö [27, 28]. pTBEV-CME contains the 5´NCR, the structural genes, and 124 bp of NS1 of Torö-2003 cloned into the pcDNA3.1 vector. The E gene in pTBEV-CME was replaced with the E gene of 93/783 using BbvCI and ApaI restriction sites to generate pTBEV-CME^93/783^. The cloning work was performed using standard molecular biology procedures and verified by sequencing (Eurofins MWG Operon, Ebersberg, Germany). Two overlapping PCR fragments comprising 5´NCR-NS1 and NS1-3´NCR of TBEV were amplified from pTBEV-CME^93/783^ and pTBEV-*luc-*rep, respectively. SP6 promoter sequence was introduced upstream 5´NCR in the first PCR fragment. Both PCR fragments were mixed by 1:1 molar ratio and 12 cycles of denaturation, annealing, and elongation were performed to generate the full-length TBEV DNA with SP6 promotor sequence. The overlapping sequence in both fragments facilitated to anneal the fragments with one another, followed by elongation with KOD KOD Hot Start DNA polymerase (Novagen®). Capped RNA transcripts of complete TBEV genome were synthesized using MEGAscript® SP6 in vitro transcription kit (Ambion) as per the manufacturer’s instructions. A mixed (1:1) population of HEK293 and VeroB4 cells was transfected with in vitro-transcribed RNA to rescue the infectious virus as previously described [29].

### RNA isolation and qPCR

RNA was isolated from cells and organs as previously described [24, 30]. Total RNA (500 ng for tissue samples and 200 ng for cultured cells) was used to synthesize cDNA using Quantitect reverse transcription kit (Qiagen) according to the manufacturer’s instructions. GAPDH, CXCL10, TNFα, Caspase3, CD45, Ly-9, and CD3 transcripts were detected by Quantitect primer assay (Qiagen) and the KAPA SYBR FAST qPCR kit (KAPA Biosystems), TBEV transcripts were detected using previously described primers and probes [31], and qPCR was run using a StepOnePlus fast real-time PCR system (Applied Biosystems).

### Viral infections, virus initial infection, and entry assay

All TBEV strains were titrated side by side to ensure that the infections were performed with the same MOI and FFU. TBEV was titrated using focus-forming assay as previously described [32]. In brief, viruses were serial diluted in 10-fold dilutions, followed by 1 h of inoculation at 37 °C and 5% CO_2_ on VeroB4 cells. After 48 h of infection, cells were fixed with 4% formaldehyde, permeabilized in PBS containing 0.5% trition-X-100 and 20 mM glycine. Virus was detected using primary monoclonal mouse antibodies directed against TBEV E (1:1000, 1786 [33]) and secondary anti-mouse HRP conjugated antibodies (1:2000, Thermo Fisher Scientific). Viral foci were then revealed by incubation with TrueBlue peroxidase substrate (KPL, Gaithersburg, MD). TROPHOS assay was performed as previously described [24, 25].

The initial infection events including binding and entry was analyzed by infecting cells with an MOI of 10 for 1 h at 37 °C 5% CO_2_, followed by three washes with PBS and before cell lysis. To analyze the entry of virions into cells, fresh medium was added after 1 h infection and cells were incubated for another 2 h at 37 °C and 5% CO_2_ before being washed with PBS-EDTA, trypsinized for 10 min, washed 3 times in PBS, and then lysed. Viral burdens were measured using qPCR and presented in relation to 93/783.

### Flow cytometry

Neurons were isolated from C57BL/6 mice at embryonic day 17 and cultured for 7 days before infection with 0.1 MOI of 93/783, Torö93E, or Torö at 37 °C 5% CO_2_ for 1 h. After 12 h of infection, cells were treated with 5 μg/mL of brefeldin A. Twenty-four hours post infection (h.p.i), cells were fixed in 4% formaldehyde. Then, permeabilized in PBS 20 mM EDTA, 2% FBS, 0.02% NaN_3_, and 0.2% Tween-20 and stained with primary antibodies monoclonal mouse antibodies directed against TBEV E (1:500, 1786) and polyclonal rabbit antibodies directed against tubulin β3 (1:500 Biolegend PRB-435P) for 60 min and secondary antibodies, donkey anti-rabbit Alexa Fluor 488 (1:2000, A21206 Thermo Fisher Scientific), and donkey anti-mouse Alexa Fluor 647 (1:2000, A31571 Thermo Fisher Scientific) for 60 min. After one wash, cells were taken up in PBS 2% FBS and analyzed with flow cytometry using a FACS LSR II instrument (Becton Dickinson). Results were analyzed using FACSDiva software (Becton Dickinson). Tubulin β3 was used to gate for neurons, and the percentage of infected cells was determined within this population.

### Serum samples

A total of 36 anonymous serum samples from TBE vaccine recipients was included in our study from the German National Consultant Laboratory for TBEV. Sera from TBE vaccinated individuals (FSME-IMMUN®, Pfizer (no. 11); Encepur®, GSK (no. 12); or a combination of FSME-IMMUN® and Encepur® (no. 13)) who, according to anamnestic information, had never suffered from TBE. All individuals had a complete basic immunization, and the last vaccine shot was more than 3 months before blood sampling. Sera were stored at − 80 °C until use.

### TBEV ELISA and focus forming reduction neutralization test

Anti-TBEV IgG levels in the sera was measured by the anti-TBE Virus ELISA “Vienna” (IgG) (EuroImmune) according to the manufacturer’s instructions; cut-off was set to OD 0.213. For focus-forming reduction neutralization test, sera were diluted 1:5 in DMEM supplemented with 100 U/mL of penicillin and 100 μg/mL streptomycin (Gibco), inactivated at 56 °C for 30 min, and were further diluted in two-fold serial dilutions in DMEM. Diluted sera were mixed with 100 FFU of 93/783, Torö, or Neudörfl and incubated at 37 °C 5% CO_2_ for 30 min and were then added to VeroB4 cells. Viral foci were revealed by focusing-forming assay. Neutralizing antibody titers were calculated as the reciprocal of serum dilution that resulted in 80% reduction of foci compared to virus control. Sera from an unvaccinated subject was used as negative control.

### Statistical analysis

Statistical analysis was performed using the GraphPad Prism Software. Unpaired *t* test was used to analyze all cell culture data, Mann-Whitney test was used to analyze the difference of viral burdens and cytokine levels in infected mice, and log-rank (Mantel-Cox) test was used to analyze the significance in survival experiments. Friedman test with Dunn’s multiple comparison test was used to analyze the different vaccine abilities to neutralize the different TBEV strains, and Kruskal-Wallis test with Dunn’s multiple comparisons test were used to analyze the effects of different vaccines on the Swedish strains. The specific tests used are indicated in the figure legends.

## Results

### 93/783 is a highly pathogenic and neurovirulent TBEV strain

**Fig. 1:**
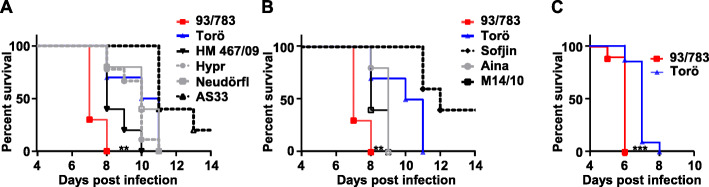
Survival analysis of mice infected with the indicated TBEV strains. Six- to 10-week-old C57BL/6 mice were infected intraperitoneally with 10^4^ FFU of TBEV strains. **a** European subtypes (HM467/09, Neudörfl, and AS33, *N* = 5, 93/783, Torö, and Hypr *N* = 10). Statistical significance between 93/783 and the other TBEV strain were calculated using log-rank (Mantel-Cox) test (***Torö, ***Hypr, **Neudörfl, ***AS33, **HM467/11). **b** Siberian and Far Eastern subtypes (Sofjin, Aina, and M14/10 *N* = 5, Torö, and 93/783 *N* = 10) compared to 93/783 and Torö. Statistical significance between 93/783 and the other TBEV strain were calculated using log-rank (Mantel-Cox) test (**Aina, **M14/10, ***Sofjin). **c** Six- to 10-week-old C57BL/6 mice were infected intracranially with 10^2^ FFU of 93/783 or Torö (*N* = 10). Asterisks indicate statistical significance vs 93/783 calculated using log-rank (Mantel-Cox) test (**P < 0.01 ***P < 0.001)

In order to identify TBEV virulence determinants, C57BL/6 mice were intraperitoneally infected with 10^4^ focus-forming units (FFU) of 6 different TBEV strains belonging to the European subtype (Fig. 1a) (Table 1). Mice were highly susceptible to all TBEV strains, and all mice died within 11 days, except for AS33 infected mice that showed 80% mortality at 13 days post infection. Mice infected with 93/783 died significantly earlier compared to mice infected with the other 5 European strains (*P* < 0.001 vs Torö, Hypr, and AS33, and *P* < 0.01 vs HM467/09 and Neudörfl, significance were calculated using log-rank (Mantel-Cox)). To further investigate the pathogenicity of 93/783, we decided to compare it to Torö, which is an infectious cDNA clone that can be genetically manipulated. Torö has a similar pathogenicity profile as other European strains (Hypr and Neudörfl) which makes it a suitable model to investigate virulence determinants. The Siberian and Far Eastern subtypes of TBEV have been associated with more severe disease compared to the European subtypes [12, 13]. To compare the pathogenicity of 93/783 and Torö to Siberian and Far Eastern TBEV strains, we infected mice intraperitoneally with 93/783 and Torö as well as two Siberian strains, M14/10 and Aina, and one Far Eastern strain, Sofjin (Fig. 1b, Table 1). Mice infected with 93/783 died significantly earlier compared to mice infected with all other strains (Sofjin *P* < 0.001, Aina and M14/10 *P* < 0.01 log-rank (Mantel-Cox) test), while Torö-infected mice survived longer compared to mice infected with the Siberian strains. Thus, 93/783 was shown to be more pathogenic than all tested TBEV strains, even those belonging to other subtypes.

**Table 1:** Description of the different TBEV strains used in the study

Virus strain	TBEV subtype	Source of isolation	Year of isolation	Country of isolation	Passage history	Reference
Torö-2003 (from now on denoted Torö)	European	*I. ricinus*	2003	Sweden	Infectious clone [29], 2 passages in VeroB4 cells	[27, 29] GenBank Accession no. DQ401140.3
HM467/09	European	*I. ricinus*	2009	Germany	3 passages in VeroB4 cells	JF501446.1 [34]
Neudörfl	European	*I. ricinus*	1970	Austria	Multiple lab passages in mouse brain and call culture	[35]
Hypr71 (from now on denoted Hypr)	European	Human blood	1953	Czechoslovakia	Multiple lab passages in mouse brain and cell culture	[36]
AS33	European	*I. ricinus*	2005	Germany	7 passages in VeroB4 cells	[37] GenBank Accession no GQ266392.1
93/783	European	Serum of a 56-year-old male	1993	Sweden	Isolation in suckling mouse brain, 4 passages in VeroB4 cells	[26] GenBank Accession no MT581212.1
M14/10	Siberian	*I. persulcatus*	2010	Mongolia	7 passages in VeroB4 cells	[38]
Aina	Siberian	Human blood	1963	Russia	Multiple lab passages in mouse brain and cell culture	[39]
Sofjin	Far Eastern	Human brain		Russia	Multiple lab passages in mouse brain and cell culture	[40]

The pathogenicity of a neurotropic virus is largely dependent on its neuroinvasiveness (ability to enter the CNS) and its neurovirulence (ability to cause disease within the CNS). To investigate the neurovirulence of the two different TBEV strains, mice were infected intracranially with 10^2^ FFU of 93/783 or Torö (Fig. 1c). Both strains were highly neurovirulent, and all mice died within 8 days of infection. There was no difference in clinical signs of disease or any difference in neurological behavior in 93/783 and Torö-infected mice. However, mice infected with 93/783 died significantly earlier than Torö-infected mice (*P* < 0.001), indicating that 93/783 is more neurovirulent. Taken together, we concluded that 93/783 is a highly pathogenic and neurovirulent TBEV strain.

### 93/783 replicates better within the brain

**Fig. 2:**
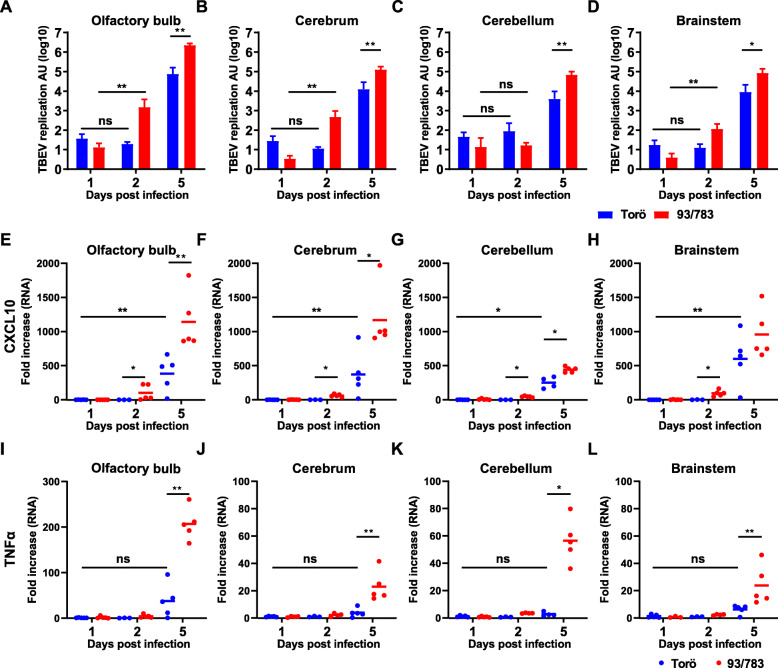
Viral burdens in brains after intracranial infection with 93/783 and Torö. Six- to 10-week-old mice were infected intracranially with 10^2^ FFU of 93/783 or Torö. Viral burdens in the olfactory bulb (**a**), cerebrum (**b**), cerebellum (**c**), and brainstem (**d**) were determined by qPCR; expression levels were normalized to the endogenous GAPDH expression and calculated using the ΔΔCT method. CXCL10 and TNFα expression levels were detected with qPCR in the olfactory bulb (**e**, **i**), cerebrum (**f**, **j**), cerebellum (**g**, **h**), and brainstem (**h**, **l**), normalized to GAPDH and expression levels in Torö day 1 for the different brain regions (*N* = 3 − 5). Asterisks indicate statistical significance calculated using Mann-Whitney test (ns = not significant, *P < 0.05, **P < 0.01)

As 93/783 was found to be more neurovirulent compared to Torö, we wanted to investigate if this correlated with increased viral replication, proinflammatory cytokines, and chemokine levels within a certain brain region. Mice were intracranially infected with 10^2^ FFU of Torö or 93/783, and different brain regions were isolated at 1, 2, and 5 days post infection (d.p.i.) (Fig. 2). Viral RNA was detected in all brain regions, olfactory bulb, cerebrum, cerebellum, and brainstem. Higher levels of viral replication in 93/783-infected mice were found in the olfactory bulb (Fig. 2a), cerebrum (Fig. 2b), and brainstem (Fig. 2d) at 2 d.p.i and in all four different brain regions at 5 d.p.i. Interestingly, viral RNA levels of 93/783 increased immediately between day 1 and day 2 in the olfactory bulb, cerebrum, and brainstem, whereas no significant increase was found in Torö-infected mice. Increased levels of the lymphocyte chemoattractant CXCL10 was detected in all brain regions day 2 post infection in 93/783-infected mice, while Torö-infected mice responded later and induced CXCL10 day 5 (Fig. 2e–h). Tumor necrosis factor TNFα RNA levels were significantly elevated day 5 for 93/783 in all brain regions, while for Torö, very low levels of TNFα was induced day 5 compared to day 1 (Fig. 2i–l). Thus, higher neurovirulence of 93/783 correlated with increased viral replication and neuroinflammation at both early and late time points.

### 93/783 possess two unique amino acid substitutions in the envelope protein which are important for neuron infection

**Fig. 3:**
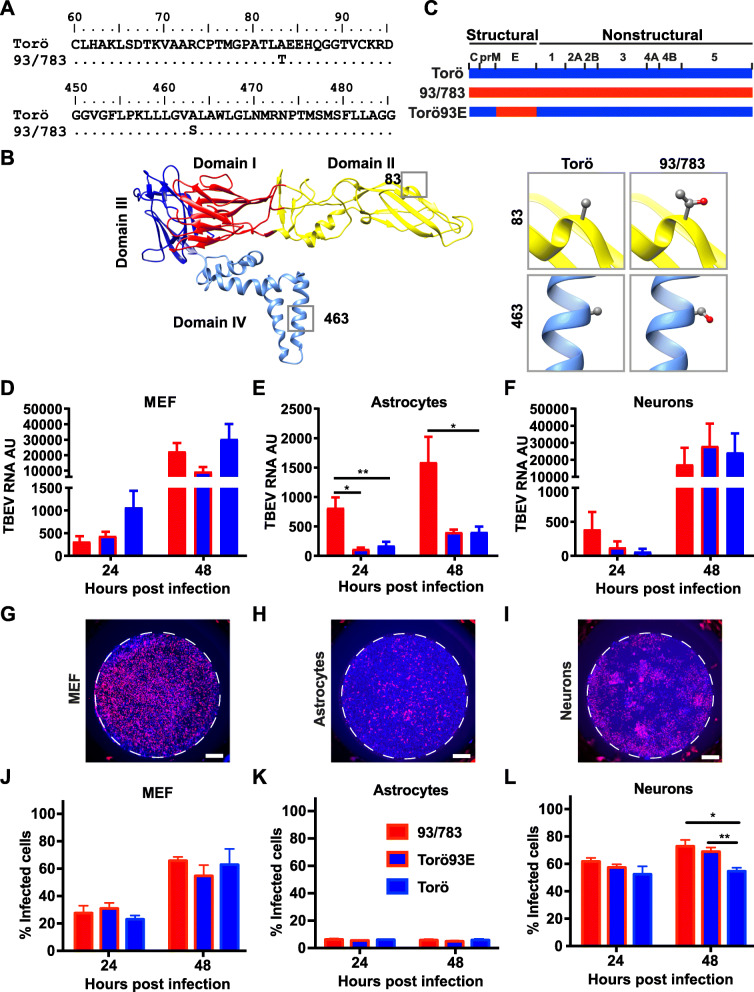
Sequence analysis, generation of chimeric TBEV virus, and infection rates in primary cells. **a** Sequence alignment of amino acid sequence of 93/783 and Torö using BioEdit software, displaying amino acid differences at residues 83 and 463; residues excluded from the alignment were the same in both strains. **b** (left panel) Single TBEV E protein monomer (PDB accession number 5O6A) showing the three extracellular domains DI (red), DII (yellow), and DIII (dark blue) and the transmembrane domain DIV (light blue). **b** (right panel) Magnification of the variations in E protein between TBEV strain Torö and 93/783 in domain II and domain IV. Residues A83 and A463 for strains Torö and T83 and S463 for strain 93/783 shown as ball and stick. Molecular graphics prepared using UCSF Chimera (https://www.cgl.ucsf.edu/chimera/). **c** The whole E protein from 93/783 was cloned into Torö in order to generate the chimeric strain Torö93E. MEFs (**d**, **g,** and **j**, MOI:0.1), astrocytes (**e**, **h**, and **k**, MOI:0.1) and cortical neurons (**f** MOI:0.001, **i**, and **l**, MOI:0.01) were infected with the indicated TBEV strains. Viral replication was determined by qPCR (**d–f**); viral RNA expression levels were calculated by normalizing to GAPDH expression using the ΔΔCT method and fold induction over the 3 h time point (input control). (g–i). Representative IF pictures of MEF, astrocytes, and neurons infected with 93/783 for 48 h, stained with anti-E monoclonal antibody (red) and Dapi, scale bar 1000 μM (**j–l**). Percentage of infected cells. Data are cumulative of two independent experiments performed in triplicates; statistical significance were calculated by parametric *t* test (*P < 0.05, **P < 0.01)

Sequence alignments of all investigated strains was performed to identify unique amino acid substitutions in 93/783 that could contribute to the increased pathogenicity. Several amino acid differences were observed in the genome; however, most of them were not unique, and found in the other less pathogenic strains, and was therefore not investigated further in this study. In the E protein of 93/783, we found two unique amino acids, which were not found in any of the other strains used for the survival experiments (Fig. 1). The alanine at residue 83 (Torö) in domain II of the E protein was a threonine in 93/783, and the alanine at residue 463 (Torö) in the stem anchor region was a serine in 93/783 (Fig. 3a–b). Residue 83 in domain II was found to be located on the surface of the virion particle, whereas residue 463 is buried in the membrane. The E protein is involved in receptor binding and membrane fusion and also constitutes the main target for neutralizing antibodies [41, 42]; thus, the E protein plays an important role in pathogenesis. To determine the function of these amino acid substitutions, a chimeric Torö virus was generated replacing the E protein with the E protein of 93/783, denoted Torö93E (Fig. 3c).

A previous study has shown that reduced TBEV growth in neuroblastoma cells correlated with attenuation and reduced neuroinvasiveness in mice [43]. Since we observed that 93/783 was more neurovirulent and replicated better within the mouse brain compared to Torö, we investigated the replication ability of the different strains in primary astrocytes and neurons, which are the most abundant cell types in the brain. Mouse embryonic fibroblasts (MEFs) were used as control to monitor viral replication in a non-CNS cell type. Cells were infected with 93/783, Torö93E, and Torö, and viral replication was determined at different time points by qPCR (Fig. 3d–f). The 3 h.p.i time point was used as an input control, and RNA levels were normalized against the input using the ∆∆CT method. The percentage infected cells was analyzed by immunofluorescent staining of the TBEV E protein (representative pictures Fig. 3g–i). The detection of the two strains by our monoclonal E protein antibody was validated in a side-by-side comparison with polyclonal anti-NS5 antibody recognizing an epitope of NS5 identical between the two strains (data not shown). In MEFs, no significant difference between the different TBEV strains was found (Fig. 3d, g, and j). In astrocytes, 93/783 replicated better compared to Torö and Torö93E at both 24 and 48 h.p.i (Fig. 3e); however, this was not due to more cells being infected (Fig. 3k). Torö and Torö93E showed very similar replication and infection efficiency, indicating that the E protein is not influencing the replication efficiency in astrocytes (Fig. 3e, k). The TBEV replication was similar for the three strains in neurons at later time points (Fig. 3f); however, we observed more neurons infected with 93/783 and Torö93E compared to Torö at 48 h.p.i (Fig. 3l); thus, the E protein of 93/783 might influence infection efficiency in neurons. Taken together, we found that the E protein of 93/783 enhanced Torö viral spread in neurons specifically, which could be of importance for pathogenesis.

### The E protein of 93/783 enhances binding, entry, and infection of neurons

**Fig. 4:**
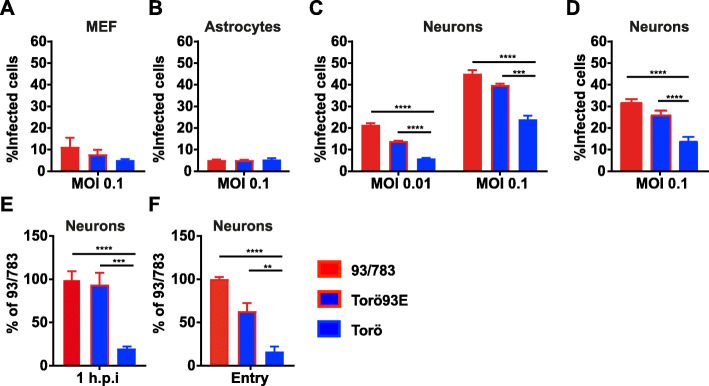
Percent cells infected during first round of infection and analysis of viral entry. Primary MEFs (**a**), astrocytes (**b**), and cortical neurons (**c**–**d**) were infected using the indicated TBEV strains. Cells were treated with 5 μg/mL brefeldin A 12 h.p.i, and 24 h.p.i cells were fixed using 4% formaldehyde and number of infected cells were determined by immunofluorescent staining of TBEV E protein followed by quantification using a TROPHOS Plate RUNNER HD (**a**–**c**) or FACS (**d**). **e** Cortical neurons were infected for 1 h (MOI:10), cells were washed and lysed and viral RNA measured by qPCR. Values are presented as % of RNA compared to 93/783, and expression was normalized to the endogenous GAPDH expression and calculated based on the ΔΔCT method. **f** Cells were infected for 1 h (MOI:10), washed, and incubated for 2 h at 37 °C. Bound but not entered, virus was removed by trypsin and cells were lysed, and RNA-quantified. Data are cumulative of at least two independent experiments performed in triplicates; statistical significance was calculated by parametric *t* test (*P < 0.05, **P < 0.01, ***P < 0.001, ****P < 0.0001)

Since the E protein of TBEV is involved in receptor binding, membrane fusion, and entry we wanted to investigate the mechanism of enhanced initial infection of 93/783. To analyze the early initial events during infection, we blocked the secretory pathway by treating the cells with 5 μg/mL brefeldin A (BFA) at 12 h.p.i before the release of new virions have started (Fig. 4a–d). Cells were fixed 24 h.p.i and stained with antibodies against the TBEV E protein. We found no significant difference in the infection rate between the different strains in MEFs or astrocytes (Fig. 4a, b). However, 93/783 as well as Torö93E infected neurons more efficiently compared to Torö (Fig. 4c, d). Since the E protein mediates receptor binding and membrane fusion, we wanted to characterize the initial binding and entry events under physiological conditions, at 37 °C. Cells were infected for 1 h and lysed for RNA extraction (Fig. 4e), or infected for 3 h followed by trypsinization (Fig. 4f) to remove bound viruses which did not enter the cell before RNA extraction. The viral burdens were measured by qPCR and were presented in relation to the 93/783 strain. The 93/783 strain showed much more efficient receptor binding and entry into neurons compared to the Torö strain. This was mediated by the E protein of 93/783 strain, because the chimeric virus showed better infection at 1 and 3 h.p.i compared to the Torö strain (Fig. 4e, f). Taken together, 93/783 was found to be a highly pathogenic TBEV strain in mice and by introducing the E protein of 93/783 into Torö, the initial infection and entry into neurons was enhanced, indicating that the amino acid substitutions A83T and A463S might be important virulence determinants by increasing neuronal infection.

### E protein of 93/783 enhances pathogenicity of Torö

**Fig. 5:**
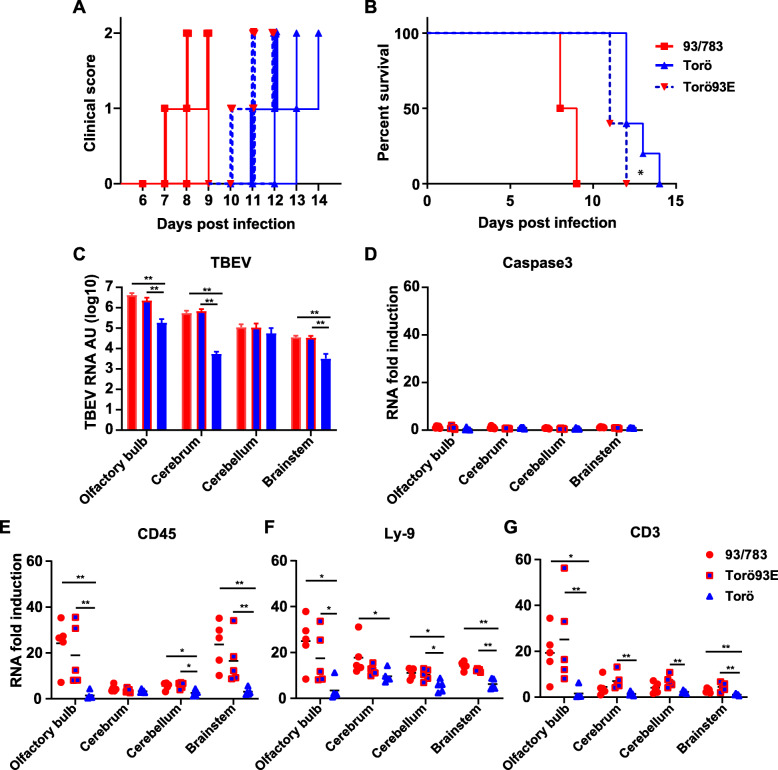
Analysis of pathogenicity, neurovirulence, and immune cell markers of mice infected with 93/783, Torö, and Torö93E. Six- to 10-week-old C57/BL6 mice were infected intraperitoneally with 10^4^ FFU using the indicated strains (93/783 *N* = 4, Torö and Torö93E *N* = 5), and clinical score (**a**) and survival (**b**) were analyzed. Statistical significance between Torö and Torö93E (*P = 0.0311), between Torö and 93/783 (**P = 0.0039) and between Torö93E and 93/783 (**P = 0.0039) were calculated using log-rank (Mantel-Cox) test. Six- to 10-week-old mice were infected intracranially with 10^2^ FFU of 93/783, Torö93E, or Torö and brain parts were isolated 5 d.p.i. Viral burdens in the olfactory bulb, cerebrum, cerebellum, and brainstem (**c**) were determined by qPCR; expression levels were normalized to the endogenous GAPDH expression and calculated using the ΔΔCT method. Caspase 3 (**d**), CD45 (**e**), Ly-9 (**f**), and CD3 (**g**) expression levels were detected with qPCR in the olfactory bulb, cerebrum, cerebellum, and brainstem; expression levels are shown as fold induction compared to mock-infected mice (*N* = 5). Asterisks indicate statistical significance calculated using Mann-Whitney test (*P < 0.05, **P < 0.01)

As we found that Torö93E infects neurons more efficiently compared to Torö, we set out to investigate if this was important for pathogenesis in vivo. Mice were infected intraperitoneally with 93/783, Torö, or Torö93E, and clinical signs was monitored over time and scored 0, 1, or 2. Mice were sacrificed when an accumulative score of 2 was reached (Fig. 5a–b). Although there were no difference in the type of clinical disease elicited by the three different strains, 93/783 showed a faster disease course compared to Torö and Torö93E (Fig. 5a). When analyzing the median survival time, we found that the 93/783-infected mice died 8.5 days post infection and Torö-infected mice survived longer with a median survival time of 12 days (**P = 0.0039 log-rank (Mantel-Cox) test). Mice infected with Torö93E died earlier compared to mice infected with Torö with a median survival time of 11 days (*P = 0.0311 log-rank (Mantel-Cox) test) (Fig. 5b).

We observed increased pathogenicity of the chimeric Torö93E compared to Torö, as well as higher viral replication in neurons in vitro. To investigate the impact of the E protein on viral replication in the brain, mice were infected intracranially with all three strains and total RNA was extracted from the olfactory bulb, cerebrum, cerebellum, and brainstem 5 days post infection (Fig. 5c–g). No differences in viral RNA levels were detected between 93/783 and Torö93E in any of the different brain parts. However, viral RNA levels of Torö as compared to 93/783 and Torö93E were lower in the olfactory bulb, cerebrum, and brain stem (Fig. 5c). To determine if neurovirulence is associated with apoptotic cell death, we measured Caspase3 RNA levels with qPCR. The TBEV infection had no impact on the mRNA level of the apoptosis marker Caspase3 in any of the brain regions (Fig. 5d).

Expression of proinflammatory cytokines like CXCL10 and TNF-α are associated with infiltration of peripheral immune cells. In order to investigate this, expression of CD45 (Fig. 5e, leukocytes), Ly-9 (Fig. 5f, B and T cells), and CD3 (Fig. 5g, T cells) were measured by qPCR. We found higher CD45 RNA levels in the olfactory bulb, cerebellum, and brain stem of 93/783- and Torö93E-infected mice as compared to those of Torö (Fig. 5e). The expression of Ly-9 and CD3 RNA increased in all brain regions upon infection, with the highest level in the olfactory bulb of 93/783- and Torö93E-infected mice (Fig. 5f–g). Taken together, these results indicate that the E protein of 93/783 affect the clinical outcome, pathogenicity, and neurovirulence of TBEV, which correlated with increased levels of viral RNA in the brain, and higher levels of infiltrating leukocytes which correspond to both T and B cells.

### TBEV vaccine neutralizes vaccine strain more efficiently compared to natural isolates

Over the years, there has been an increase in TBE vaccine breakthroughs in Sweden [14, 18], but the underlying mechanisms are not fully known. Neutralizing antibodies against the E protein play an important role in controlling TBEV infections. We hypothesized that naturally circulating Swedish TBEV strains may not be neutralized equally efficient by vaccine-derived antibodies due to the detected amino acid differences in exposed regions of the E protein known to interact with neutralizing antibodies.

**Fig. 6:**
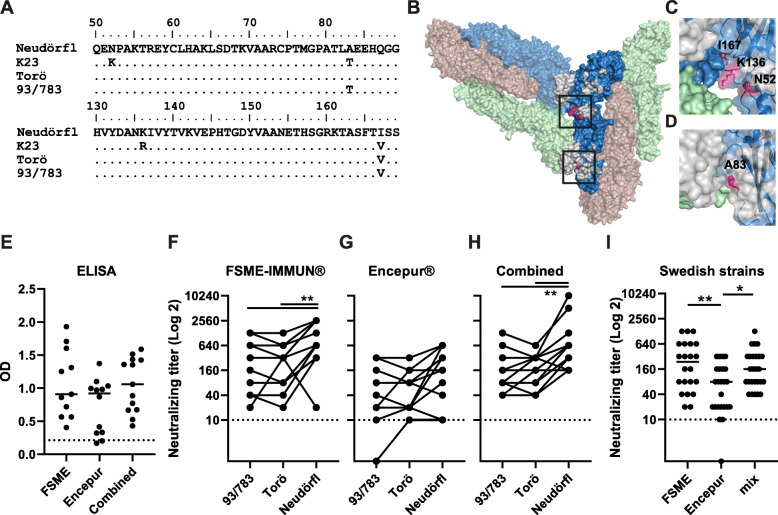
Sequence alignment. Structure of the E protein tries to explain the differences in neutralizing ability between the two vaccines. **a** Sequence alignment of the two vaccine TBEV strains Neudörfl (FSME-IMMUN®, reference) and K23 (Encepur®) with 93/783 and Torö using BioEdit software. **b** Two asymmetric units of the TBEV virion. Proposed antibody interacting sites [42] marked in grey and amino acids variations within the sites highlighted in pink. Magnification of neutralizing antibody interacting site close to the 3-fold symmetry axis (**c**) with the amino acid N52, K136, and I167 shown in pink, and the 5-fold symmetry axis (**d**) with the amino acid A83 shown in pink. PDB accession number 5O6A. Molecular graphics and structure editing were prepared using UCSF Chimera (https://www.cgl.ucsf.edu/chimera/) and PyMol (https://pymol.org/2/). **e** Anti-TBEV IgG ELISA of sera from individuals vaccinated with either FSME-IMMUN® (no. 11), Encepur® (no. 12) or a combination of both vaccines (no. 13), threshold set to < 0.213 dotted line. Neutralizing antibody titers against 93/783, Torö, and Neudörfl from individuals vaccinated with either FSME-IMMUN® (**f**), Encepur® (**g**), or a combination of both vaccines (**h**). Dotted line indicates threshold for neutralizing antibody titers. **i** Comparison of neutralizing antibody titers against 93/783 and Torö in vaccinated individuals with the different vaccines. Statistical significance was calculated using Friedman test (**f**, **h**) and Kruskal-Wallis multiple comparison test (**i**)

Sequence alignment of the E protein was performed, comparing amino acid differences among the vaccine strains Neudörfl (FSME-IMMUN®) and K23 (Encepur®) and the Swedish strains Torö and 93/783 (Fig. 6a). We found 5 amino acids in the E protein which differed between the strains. The amino acid substitution A83T, which was unique in 93/783 among the strains used in our survival analysis, was also found in the vaccine strain K23. K23 also harbored two unique amino acid substitutions N52K and K136R which were not found in Torö, Neudörfl, or 93/783, whereas Neudörfl possessed one unique amino acid substitution V167I compared to the other strains. The A463S amino acid substitution was only found in 93/783 (data not shown). One of the amino acid substitutions in 93/783, A83T, was found close to the interface between E protein and neutralizing antibodies (Fig. 6b, d) [33, 42]; thus, this substitution might be important for neutralization.

To test our hypothesis, we performed focus-forming neutralization test on sera from 36 vaccinated individuals. The individuals were vaccinated either by FSME-IMMUN® (based on Neudörfl strain, no. 11), Encepur® (based on K23 strain, no. 12), or a combination of both vaccines (no. 13). No significant difference between the IgG levels, as measured by ELISA, could be detected between the different vaccines (Fig. 6e). The neutralization efficiency was tested against the two Swedish strains 93/783 and Torö as well as the vaccine strain Neudörfl (Fig. 6f–h). The human sera from individuals vaccinated by Neudörfl-based FSME-IMMUN® or a combination of both vaccines (Fig. 6f, h) neutralized Neudörfl significantly better compared to the Swedish isolates Torö and 93/783. Since V167I is the only difference between Neudörfl and the Swedish strains in the E protein, this indicates that this residue might contribute to the difference in neutralizing antibody titers (Fig. 6b, c). We also found that FMSE-IMMUN® or a combination of both vaccines neutralized the Swedish strains Torö and 93/783 more efficiently compared to Encepur® (Fig. 6i). This might be due to the fact that the E protein of the Swedish strains is more similar to Neudörfl compared to K23 (Fig. 6a). Despite this, both FSME-IMMUN® and the combination of both vaccines resulted in sera with neutralizing activity above the threshold for what is considered neutralizing titers. The sera from K23-based Encepur® vaccine showed overall lower neutralizing titers to all three strains tested (Fig. 6g, i), with some sera not reaching to neutralizing titers, but there were no significant difference between the strains (Fig. 6g). This indicates that the residue N52K is important for generating a neutralizing response against several different viruses, in accordance to previous studies [44]. Structurally, this amino acid is found in a previously identified binding site of a neutralizing antibody (Fig. 6b–c) [42]; thus, genetic divergence resulted in reduced neutralizing antibody titers and might be a contributing factor to vaccine breakthroughs.

## Discussion

Here we identified novel virulence determinants contributing to disease severity in TBEV infection. By screening different TBEV strains in mice, we identified one highly pathogenic European strain, 93/783. 93/783 is a low passage strain isolated from the sera of a human TBE patient in Sweden, who later developed encephalitis [26]. The human isolate 93/783 has been under selection pressure of a mammalian immune response, whereas the other strains are either tick isolates (Torö, M14/10, HM467/11) or reference strains with high passage number in suckling mouse brain and cell culture (Hypr, Neudörfl, Aina, and Sofjin). This might be one factor contributing to the high virulence of 93/783, as passaging of TBEV in cell culture has previously been reported to result in attenuation in vivo [22, 45]. However, when we analyzed the prevalence of the T83 residue in other TBEV strains deposited in GenBank (using: www.viprbrc.org) we found only 7 TBEV sequences (GenBank no: AM600965.1, M94956.1, KT224355.1, AB049353.1, AB049350.1, AF091010.1, and MH021184.1) out of more than 1000, indicating that it is a quite rare amino acid at this position. Furthermore, the viruses carrying this rare amino acid in position 83 are isolates from both ticks and patients [46, 47] and belong to different subtypes, suggesting that it is neither a common mammalian adaptation of the virus, nor a specific trait of a certain subtype.

We found 93/783 to be more neurovirulent and induce more neuroinflammation markers compared to Torö. An immediate increase in viral replication and the chemokine CXCL10 were observed in the brains intracranially infected with 93/783 mice, whereas viral replication and CXCL10 were only detectable 5 d.p.i in Torö-infected mice. Interestingly, a marked increase of TNFα was observed for the highly pathogenic 93/783 5 days post infection, but this increase was absent in Torö at the same time point. Faster spread and higher viral RNA load might be due to more infected cells in the brain. This correlated with stronger neuroinflammation and faster disease development and could be important determinants of pathogenesis. The E protein is involved in receptor binding and membrane fusion and could be of great importance for fast spread and infection within the brain. Apart from its role in receptor binding and membrane fusion, the E protein also constitutes the most important antigenic structure [41]. Previous studies have indicated that the E protein is important for pathogenesis; amino acid changes in the E protein which resulted in increased net positive charge showed lower pathogenicity and neuroinvasiveness in vivo [22, 45, 48]. The increase in positive charge results in stronger binding to glycosaminoglycans which is an advantage in vitro, but reduces the neuroinvasiveness of the virus in vivo by impeding the virus ability to spread from cell to cell [48]. Similar findings have been observed for mosquito-borne flaviviruses [49]. Furthermore, an antibody escape mutant, containing a Y384H mutation in the E protein, also resulted in reduced neuroinvasivness [50], demonstrating the important role of the E protein in TBEV pathogenesis. To test the importance of A83T and A463S amino acids, a chimeric Torö93E virus was generated, by transferring the E gene from 93/783 into Torö. We found that the E protein of 93/783 mediated better binding and infection into neurons, and since the A83T amino acid substitution is located on the surface, it is more likely that this specific amino acid contributes to this effect. Interestingly, we saw no difference between the strains during the infection in MEFs, indicating that the effect of A83T residue on virus entry and infection is cell-type-specific for neurons. In astrocytes, no difference in number of infected cells were detected between the strains, and this might be due to the low number of infected cells and the rare event of TBEV-infected astrocytes [30, 51–54]. The low infection rate could be explained by the fact that the astrocytes induce a rapid type I interferon response that prevents the infection of the neighboring cells [25]. However, 93/783 replicated more efficiently in astrocytes compared to Torö, whereas the chimera virus behaved like Torö. This could be explained by amino acid differences in other parts of the virus, both in other structural as well as non-structural (NS) proteins, e.g., NS3 and NS5, as shown in other studies [55], which could contribute to pathogenesis, and these differences will be further investigated.

Importantly, we found that the differences in neuron-binding and infection in vitro between the different TBEV strains correlated with the pathogenicity and neurovirulence in vivo. After intracranial infection, the chimeric Torö virus containing the E from 93/783 replicated better compared to Torö. In addition, immune cells infiltrated to a greater extent into the brains infected with these viruses. Infiltrating T cells and specifically CD8^+^ T cells have been reported to mainly cause immunopathology, whereas CD4^+^ T cells play a protective role during TBEV infection [56]. We found higher levels of CD3 RNA in the chimera virus as compared to the lower pathogenic Torö virus in all brain regions, which probably correspond to higher levels of cytotoxic T cells that might enhance the neuropathogenicity and help to explain the increased neurovirulence.

There are two commercially available vaccines against TBEV in Europe. These are considered to be safe and efficacious; however, vaccine breakthroughs occur [14]. In a retrospective study concerning reported TBEV cases in the Stockholm County in Sweden during 2006 to 2015, it was found that 81% of vaccine failure patients were over 50 years old and 26% of the patients had diseases which involved the immune system; the mortality rate was 6% [57]. The exact mechanisms behind the vaccine breakthroughs are not known, but the immune response and the genotype of the patient as well as the genetics of the viral strain might contribute.

Here we wanted to investigate if natural variation in the E protein could affect the efficiency of neutralization by the TBEV vaccines. When we compared the amino acid sequence of the E protein of Torö, 93/783, and the two vaccine strains Neudörfl and K23, we found that the amino acid differences among the strains were in previously identified neutralizing epitopes, and neighboring residues had been found to affect neutralization [42]. To investigate the role of these amino acid differences for neutralizing antibody titers, we tested the sera from individuals vaccinated with either FSME-IMMUN®, Encepur®, or a combination of both vaccines. Although there were no significant difference in IgG titers, we found that sera from FSME-IMMUN® vaccinated patients possessed higher neutralizing titers against the vaccine strain Neudörfl compared to the natural isolates Torö and 93/783. Neutralizing antibodies have been shown to be induced by both FSME-IMMUN® and Encepur®, and vaccine-induced protection against TBEV has been shown to be mediated by 497 antibodies against the E protein [58–60]. The only difference in amino acid sequence of the E protein of Torö and Neudörfl is the I167V which could be important for neutralization and explain the higher neutralizing titers against Neudörfl in FSME-IMMUN® vaccinated patients.

There was no significant difference in neutralizing titers against Torö, 93/783, or Neudörfl in the sera of Encepur® vaccinated individuals. Interestingly, FSME-IMMUN® was shown to neutralize Swedish strains better compared to Encepur®, where some sera showed borderline neutralizing titers after Encepur® vaccination and one serum did not neutralize 93/783 at all. A previous study found that the amino acid K52 is very important for efficient neutralization by Encepur®. This amino acid is found in the K23 vaccine strain (GenBank accession no. AM600965.1) [44]; however, this is the only TBEV strain found in the database (GenBank) sequenced that contains this amino acid in that position (www.viprbrc.org). This could also explain why the Swedish isolates which lacks the K52 are not neutralized as efficiently by Encepur®. Thus, genetic divergence from the vaccine strain might reduce neutralizing antibody titers and might be a risk factor for vaccine breakthroughs.

## Conclusions

In summary, we identified two unique amino acids in the E protein of a highly pathogenic and neurovirulent TBEV strain, which enhanced TBEV pathogenicity, neurovirulence, and viral replication in the brain in mice. Most likely by specifically increasing TBEV entry and infection of neurons, TBEVs primary target cell in vivo. In contrast, this amino acid (position 83) is not directly involved in determining the neutralization capability of the vaccines FSME-IMMUN® and Encepur®. However, lower neutralizing titers against the Swedish strains was observed with the Encepur® vaccinated sera, compared to FSME-IMMUN®, probably because of position 52 in the vaccine strain K23 (Encepur®). Sera from FSME-IMMUN® and combined vaccination showed overall higher neutralizing titers against the Swedish strains. Since these observations are from a relatively small sample size, they need to be followed up in order to give clear advice about vaccinations.

## Data Availability

All data and materials is available upon request.

## Abbreviations

CNS: Central nervous system
h.p.i: Hours post infection
d.p.i: Days post infection
BFA: Brefeldin A
E: Envelope
MEF: Mouse embryonic fibroblast
qPCR: Quantitative reverse transcription PCR
PBS: Phosphate-buffered saline
FFU: Focus-forming unit
WT: Wild-type
TBEV: Tick-borne encephalitis virus
